# Surface geoenvironments shape organic matter inputs in iron-ore caves of Carajás, southeastern Amazonia

**DOI:** 10.1371/journal.pone.0344450

**Published:** 2026-03-11

**Authors:** Luiza Santos Reis, José Tasso Felix Guimarães, Cecília Yuki Gomes de Sá, Enrico Bernard, Luiz Carlos Ruiz Pessenda, Markus Gastauer, Paulo Eduardo de Oliveira

**Affiliations:** 1 Instituto Tecnológico Vale, Belém, Brazil; 2 University of São Paulo, Institute of Geosciences, São Paulo, Brazil; 3 Universidade Federal de Lavras, Lavras, Brazil; 4 University of São Paulo, Center for Nuclear Energy in Agriculture, Piracicaba, Brazil; 5 The Field Museum of Natural History, Chicago, Illinois, United States of America; University of Siena, ITALY

## Abstract

Cave ecosystems rely heavily on organic matter inputs from the outside to support their trophic networks and biodiversity. In the iron-ore caves of Carajás, southeastern Amazonia, bat guano is a major source of organic matter and integrates signals from the surrounding landscape. Nevertheless, our understanding of the relative contributions from other surface-derived organic matter sources, and how these organic material sources are transported into cave environments is limited. Through a combination of carbon and nitrogen isotope analysis, carbon-to-nitrogen ratio, Bayesian mixing models, and compositional data analysis, the present study estimated for the first time, the contribution of different geoenvironmental units of ferruginous outcrops (*canga*) to cave deposits in four iron-ore caves of Carajás on a preliminary scale. The results indicate a consistent contribution of organic matter derived from poorly drained depressions, particularly swampy fields (median: 41.5–42.9%) and grasslands (median: 8.8–15.1%). These patterns suggest that water infiltration via fractures is an important pathway connecting the surface environment and underground systems, thereby contributing alongside the accumulation of bat guano. This study demonstrates the ecological importance of caves as integrative components of the surrounding landscape and further emphasizes the need to conserve both cave ecosystems and the surface ecosystems that support them by quantifying surface–subsurface connectivity.

## Introduction

Caves are distinctive fragile ecosystems which mainly depend upon allochthonous organic matter (OM) for biodiversity and ecological processes [1,2]. OM is introduced in dry terrestrial cave systems mainly through bat guano, plant debris, and inorganic detritus transported from the surface [3]. Bat guano, especially, acts as a nutrient-rich substrate facilitating decomposers, detritivores, and other organisms important to the subsistence of cave ecosystems [4]. In caves that are affected by water flow, such as intermittent or perennial streams, the OM inputs are primarily controlled by water – it transports organic and inorganic material from above to within the cave system. In both cases, these inputs form the primary energy source for many cave-dwelling organisms and underpin subterranean food webs [5].

Caves are not disconnected or isolated systems – they are part of a much larger system, where matter and biota flow continuously between the surface and subterranean environments [3]. The input of organic matter, water, mineral particles and organisms into caves often reflects the ecological and climatic conditions of the surrounding landscape [6]. Cave compartments – such as speleothems, guano, or cave soils – can be used as paleoenvironmental archives that retain information regarding past climates [7], vegetation changes [8], and anthropogenic impacts [9]. Besides, cave CO₂ and isotopic variation has been associated with external temperature, vegetation cover, and hydrological inputs [10]. These examples emphasize the importance of studying the material exchange process between the surface and cave systems.

Iron-ore caves have been documented in the southeastern Amazonia of Carajás [11] and are frequently surrounded by a variety of vegetation types, including forests and rupestrian savanna, in the naturally open canga outcrops [12]. These caves contain large bat populations [13] and receive significant guano input [14]. Such deposits may be mixed with detritus from weathering saprolite and iron crust, along with surface runoff carrying soil particles outside the caves. Nevertheless, the exact surface sources contributing to cave deposits and their relative proportions remain quantitatively unassessed. The relative contribution of surface ecosystems to cave deposits is important in understanding surface–subsurface connectivity and karst ecosystem functioning. Alteration of surface vegetation cover and land use directly impacts sediment input, organic matter transport, and nutrient availability in cave environments, affecting biodiversity and biogeochemical processes. Therefore, this study provides a clear understanding of how regional ecosystem dynamics are recorded in subterranean archives and how environmental changes at the surface may impact cave systems.

This study seeks to deepen knowledge on OM deposition in cave deposits in iron-rich tropical areas. To fill this information gap, the study uses carbon (δ¹³C) and nitrogen (δ^15^N) isotope analysis, carbon-to-nitrogen ratio (C:N), and a Bayesian mixing model (MixSIAR) [15] to calculate the contributions of distinct geoenvironments to allochthonous OM in Carajás cave deposits. δ¹³C corresponds to the photosynthetic pathways of source vegetation and aids in separating C₄ plants (grasslands) from C₃ plants (forest patches and rupestrian savanna) [16]. The higher δ¹³C values (mean of –28.3‰) can be attributed to adaptations to periodic drought phenomena, which may result in rupestrian savanna yielding higher δ¹³C values relative to rainforests (mean of –32.0‰) [17]. High δ^13^C values are not found in forest formations (with low water shortage or with high rainfall) which often have lower values of δ^13^C [16]. The ratio of OM obtained from plant (usually high C:N) and soil/microbial (typically low C:N) sources is one more metric available for OM separation [18]. Such as shrublands with depleted δ^13^C [19] will also be similar to the forest vegetation but their higher C:N ratio further discriminates between these sources. Using canga environment caves on the Carajás plateaus (eastern Amazonia) as a case study, we (i) examined the distribution of δ^13^C and δ^15^N values, besides C:N ratios along cave deposits followed by (ii) implementing a Bayesian isotope mixing model (MixSIAR) to quantify relative contributions of OM sources derived from surrounding landscapes and (iii) assessing the deposits of Fe, Ti, and P to determine their respective roles of detrital inputs and in situ biogenic production in cave deposit accumulation.

## Biogeographic background of the study region

### Location, climate and vegetation

**Fig 1:**
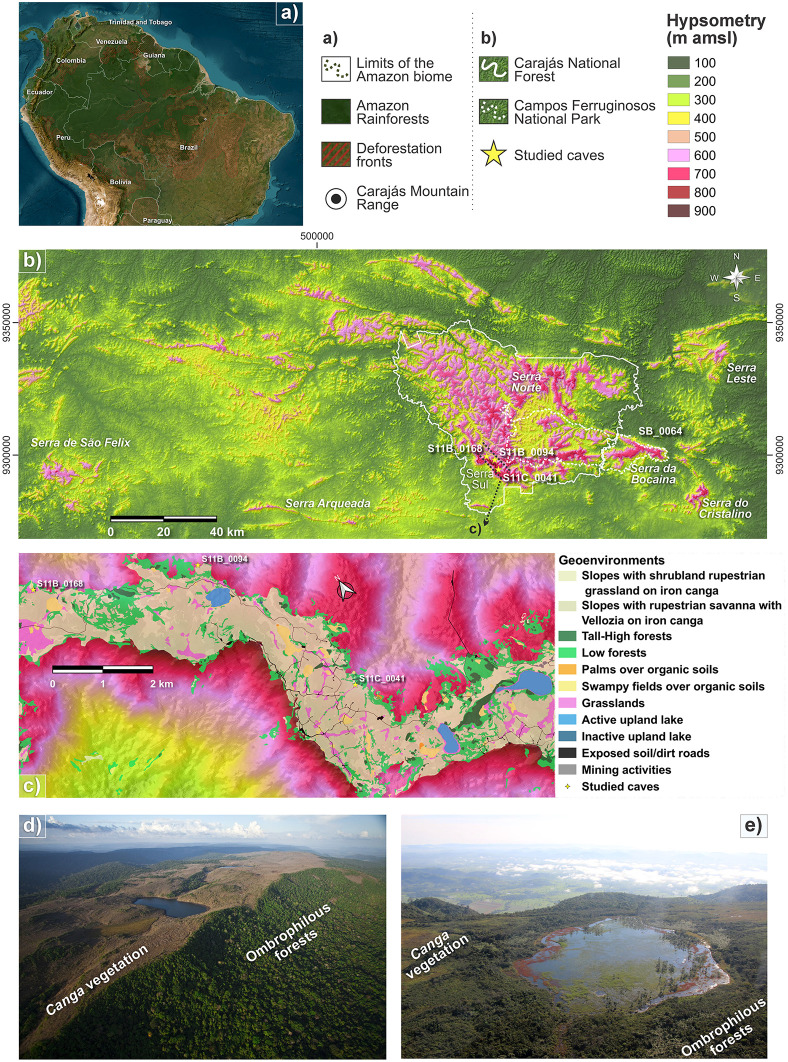
Location of study sites. **(A)** Study area within the context of South America. **(B)** Hypsometric map indicating the studied caves (yellow stars): Serra da Bocaina – SB_0064; Serra Sul – S11B_0168, S11B_0094, and S11C_0041. **(C)** Spatial distribution of geoenvironmental units across the Serra Sul plateau. **(D)** Aerial view of Serra Sul. **(E)** Aerial view of Serra da Bocaina.

The study site is situated in the Carajás National Forest (CNF; 05°52′11″S–06°32′13″S, 49°53′28″W–50°44′29″W), and Campos Ferruginosos National Park (CFNP; 06°19′46″S–06°23.09′S, 49°48′19″W–50°02′15″W), two contiguous protected areas in southeastern Brazilian Amazonia (Fig 1a, b). Although these areas are considered protected, increasing deforestation of surrounding tropical forests poses a growing threat to them.

Based on regional geology, the CNF and CFNP are inserted in the Carajás Basin, a rift-related basin composed of mafic to felsic volcanic sequences and banded iron formations of the Grão Pará Group [20,21], as well as metavolcanic acidic to metabasic rocks and metavolcanoclastic rocks of the Igarapé Cigarra Formation [21]. These were largely weathered during the Cretaceous-Cenozoic transition under a paleotropical climate, inducing deep immature lateritic profiles and medium-altitude lateritic plateaus [22]. The upper alteration mantle forms a canga (surface crust) which constitutes the regional plateaus in the Serra dos Carajás. At the transition from the duricrust to the underlying saprolite or even within the duricrust, porous and permeable ‘low-density zones’ and hollows formed as a result of the interaction of Fe- and Al-rich percolating solutions and groundwater flow [23]. As time passes, a variety of caves are exposed to the surface through the collapse and erosion of plateaus and nearby terrain. Currently, dripping is prevalent in these caves, due to their strong connectivity with the surface, the presence of fault and fractures, and the intrinsic porosity of *canga* [24].

**Fig 2:**
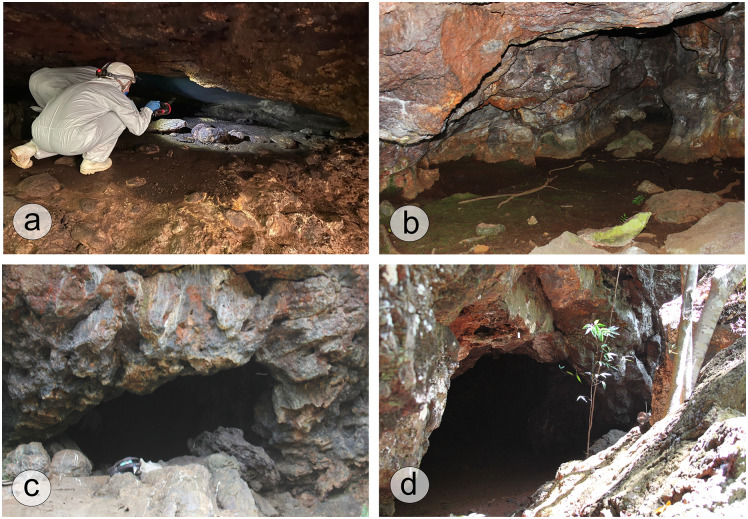
Photographs of the studied caves in the Carajás region, southeastern Amazonia. Caves: **(a)** SB_0064, **(b)** S11C_0041, **(c)** S11B_0168, **(d)** S11B_0094. Source: first author’s archive.

The caves are located in Bocaina and the Serra Sul plateaus (Fig 1c, d; Fig 2a-d) at elevations ranging from 600 to 800 m [25]. They can be mostly found at the base of escarpments, and vary among various landscape conditions including lake shores, plateau-edge escarpments, and colluvial slopes situated along ridges [14]. The area has over 1,500 cavities, many of which are shallow and short-length caves (approximately 30 m long) [14].

The region experiences two well-defined seasons. In the rainy season (November to May) the total rainfall is between 1545 mm and 1863 mm while in the dry season (June to October) it is between 159 mm and 321 mm [26]. With an average annual temperature of 27.2°C, January has a minimum of 26.6°C, and September has a maximum of 28.1°C [27]. Currently, regional vegetation is predominantly composed of forest formations, mainly humid evergreen tropical forests (HETF) and semi-deciduous forests (SDF) [28–30].

**Fig 3:**
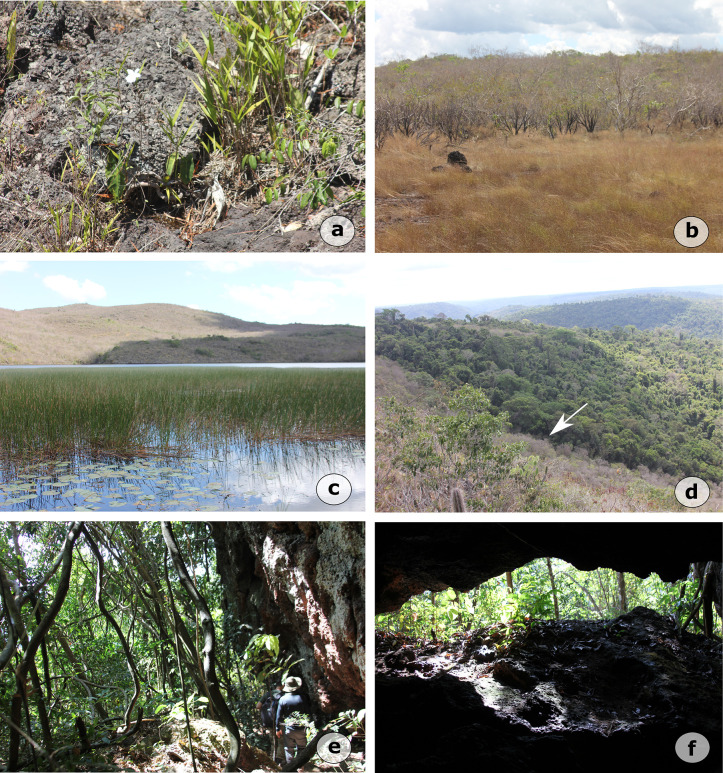
Vegetation types in Serra Sul de Carajás and Serra da Bocaina, southeastern Amazonia: **(a)** Open montane savanna on ferruginous outcrops; **(b)** Grasslands with *Vellozia* fields; **(c)** Swampy areas during the rainy season; **(d)** Ecotone between rupestrian savanna and ombrophilous forest on plateau slopes; **(e)** Open ombrophilous forest, commonly found near cave entrances; **(f)** View of the entrance to cave S11B_0094.Source: first author’s archive.

A mosaic of open and woody vegetation types is directly associated with *canga* landscapes (Fig 3a, b) with unique types of forms such as grasslands (seasonally inundated canga), scrublands, and swamps (Fig 3c) dominated by macrophytes [29,31]. Small forest patches occasionally interrupt rupestrian savanna, forming where shallow soils accumulate. The HETF, developed on thicker soils along plateau slopes (Fig 3d), comprise both dense and open ombrophylous forests (Fig 3e). Dense ombrophylous forests exhibit high species richness, particularly in Fabaceae, Moraceae, and Sapotaceae, and include emergent trees that may exceed 50 m in height [32]. Open ombrophylous forests, on the other hand, are sparser and typically contain a greater abundance of palms and lianas [32].

The vegetation cover is distributed in four important geoenvironments [33], which manifest differentiated pedological, geomorphological and geological features: (1) slopes with rupestrian savanna, (2) slopes with patches of forest, (3) poorly drained depressions, and (4) upland lakes (Table 1).

**Table 1:** General characterization of the geoenvironmental units of the Serra Sul plateau, in Carajás National Forest.

Landscape compartments	Geoenvironmental units	Pedoenvironments	Vegetation
*Slopes with rupestrian savanna*	Slopes with rupestrian savanna with *Vellozia* on iron canga	Shallow, well-drained soils rich in organic matter over a continuous canga layer, with an abundance of termite mounds	Shrub-herbaceous stratum with an abundance of the genera *Axonopus*, *Vellozia*, *Sobralia*, *Ipomoea*, and *Andropogon*
	Slopes with shrubland rupestrian savanna on iron canga	Shallow, well-drained soils with pockets of organic matter in the fractures of the canga layer, abundant termite mounds	Shrub stratum with an abundance of the genera *Callisthene*, *Byrsonima*, *Bauhinia*, *Alibertia*, and *Mimosa*, which are characteristic of this phytophysiognomy
*Slopes with forest patches*	Tall forests on deep soil over degraded canga	Deep, well-drained soils with a significant amount of canga fragments (concretions) in sand and silt sizes. High levels of P (phosphorus) when near caves	Tree stratum with abundance of the genera *Pouteria*, *Sacoglottis*, *Myrcia*, *Miconia*, *Ficus*, and *Cupania*
	Low forests over intermediate soils of degraded ironstone canga	Deep, well-drained soils with a significant amount of canga fragments (concretions) in sand and silt sizes	Ecotonal formation with genera common to the geoenvironments: high forest patches, transition forest, and shrubby rupestrian savanna
	High forests of transition over deep soils of degraded ironstone canga	Deep, well-drained soils with a significant amount of canga fragments (concretions) in sand and silt sizes	Tree stratum with abundance of the genera *Mouriri*, *Caryocar*, *Eugenia*, *Casearia*, *Guatteria*, *Eugenia*, and *Myrcia*
*Poorly drained depressions*	Grasslands, moderately drained, over nodular ironstone canga	Shallow, moderately drained soils, rich in organic matter, over a continuous layer of ironstone canga	Graminoid stratum, with predominance of the families Xyridaceae, Cyperaceae, Poaceae, and Eriocaulaceae
	Swampy fields over organic soils	Organic soils, very deep, poorly drained, in an environment with significant accumulation and conservation of organic matter	Lacustrine vegetation with occurrences of the families Cyperaceae, Eriocaulaceae, Nymphaeaceae, Iridaceae, Lentibulariaceae, Xyridaceae, with abundance of Poaceae and ferns, as well as aquatic macrophytes
	Buriti palm over organic soils	Organic soils, deep, poorly drained, in an environment with significant accumulation and conservation of organic matter	Shrub layer with dominance of the species *Mauritia flexuosa*, *Mauritia carana*, and herbaceous species of the grasslands
*Upland lakes*	Submerged vegetation of lake margins	–	Aquatic environment with Cyperaceae, Eriocaulaceae, *Typha* sp., *Eleocharis* sp., as well as other macrophytes
	Permanent lakes	–	Macrophytes

### Organic matter sources in cave deposits

Cave deposits are usually a blend of organic and inorganic materials that accumulate over time, driven mainly by biological activity within and around the cave and transport of allochthonous matter into its interior [3]. In dry caves, at least six potential sources of organic material have been reported: (a) *Bat guano* – one of the most prominent organic materials found in caves, especially those with a large population of bats [14], and may consist of insect remains, fruit seed or pulp, or even blood, for example in caves containing hematophagous bats. (b) *Animal remains* – caves frequently act as shelters for animals, including rodents, birds, reptiles and larger mammals [34].

Over time, these animals may eventually die inside the cave, leaving carcasses, skeletal remains, fur, and other organic materials behind. These remains break down, contributing to the organic matter in the cave deposits [35]. Predators may also leave behind bones or other remnants of their prey. (c) *Plant matter* – while caves are not typically environments where plants grow, plant matter can still accumulate through external sources [36]. For example, vegetation near cave entrances may be blown or washed into the cave by wind or water. This plant material, such as leaves, twigs, and seeds, can contribute to the organic content in cave deposits. (d) *Fungal and microbial activity* – caves support unique ecosystems of fungi [37] and microorganisms [5], many of which thrive on the organic matter present, such as guano or decaying animal and plant material. These microbes further contribute to the breakdown of organic materials and generate new organic compounds in the process. Fungi are known to develop in environments rich in guano [38] or decaying organic matter. (e) *Human activity* – in certain caves, human activity can be a significant source of organic matter. Archaeological sites might have food scraps, charcoal from fires, and human skeletons [39]. Found in caves used by humans for shelter or as ceremonial sites, the organic debris in these caves can accumulate over centuries [40]. (f) *Insect remains and activity* – because caves often sustain large numbers of insects – including beetles, cockroaches, and cave crickets – these can also contribute to organic matter, producing dead insects, molted exoskeletons, and feces. In caves, these insects are commonly linked to their food sources, taking up organic matter such as guano [5], which can yield further organic byproducts. In caves with active water flow, such as those influenced by streams or groundwater, water serves as a major vector for the transport of allochthonous materials such as soil particles, plant debris, and microorganisms. In areas characterized by reduced or intermittent flow velocity, these inputs can accumulate over time and allow organic matter to be deposited into cave deposits. In addition, flooding can introduce a high quantity of material in short spans of time and thereby can lead to significant deposition of organic material during high-energy hydrological pulses. The organic matter also decomposes at a wide range of stages based on environmental parameters such as temperature [41], humidity [42], as well as the oxygen level [43]. Organic decomposition happens more gradually for long durations in low oxygen conditions like swamps or ponds. In some caves, with large bat populations, bat guano accumulates quickly, which leads to bat guano-rich cave deposits [44,45]. However, among sites with greater microbial or fungal populations, organic matter can be degraded more quickly [46].

## Materials and methods

### Core sampling

**Fig 4:**
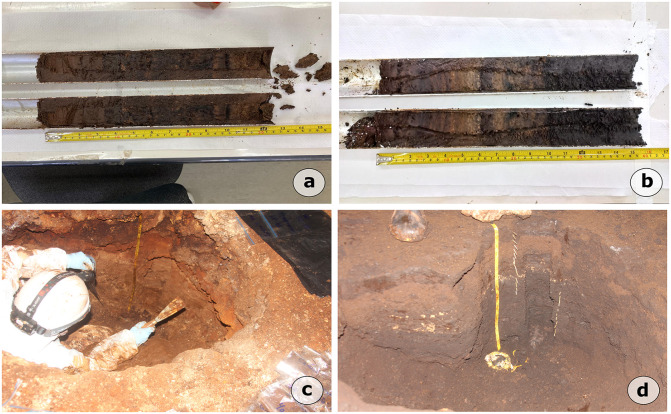
Photographs of cave deposits collected from iron-ore caves in the Carajás region. Caves: **a)** S11B_0168, **b)** SB_0064, **c)** S11B_0094, **d)** S11C_0041. Source: first author’s archive.

On the basis of research permit n° 82199−1 issued by MMA/ICMBio/SISBIO, fieldwork at Carajás was conducted between June 27 and July 13, 2022. Based on the presence of guano deposits, four caves were selected: one situated in Serra da Bocaina (SB_0064) and three found in Serra Sul de Carajás (S11B_0094, S11B_0168, S11C_0041). The sampling methods differed according to the deposit characteristics. In caves SB_0064 and S11B_0168 which contain unconsolidated deposits, cores measuring 35 cm and 28 cm were collected by stainless steel tubes, respectively. In contrast, trenches were excavated in caves S11B_0094 (101 cm deep) and S11C_0041 (52 cm deep), where the deposits were more consolidated (Fig 4; Table 2).

**Table 2:** Location and main characteristics of studied caves in Carajás region.

Caves	Municipality, State	Latitude	Longitude	Vegetation type	_Site description_
SB_0064	Serra da Bocaina, Canaã dos Carajás, PA	06°16’36.72“S	_49°55’3.76“W_	Open ombrophylous forests	Type: bat cave Situated on plateau slopes, at the savanna-forest transition; cave predominantly colonized by a large number of insectivorous bat species (*Pteronotus gymnonotus* and *Pteronotus personatus*)
_S11B_0094._	_Serra Sul,_ _Canaã dos Carajás, PA_	_06°20’56.63“S_	_50°23’36.59“W_	Open ombrophylous forests	Type: non-bat cave Situated on plateau slopes, at the savanna-forest transition; cave colonized by small groups of different bat species
_S11B_0168._	_Serra Sul,_ _Canaã dos Carajás, PA_	_06°20’13.80“S_	_50°25’15.56“W_	Open ombrophylous forests	Type: bat cave Situated on plateau slopes, at the savanna-forest transition; cave predominantly colonized by a large number of insectivorous bat species (*Pteronotus gymnonotus* and *Pteronotus personatus*)
_S11C_0041._	_Serra Sul,_ _Canaã dos Carajás, PA_	_06°22′56.56″S_	_50°22′50.87″W_	Open ombrophylous forests	Type: non-bat cave Situated on plateau slopes, at the savanna-forest transition; cave colonized by small groups of different bat species

### Stable isotopes and elemental analyses

A total of 168 bat guano samples (6–50 mg) were taken at 1–4 cm intervals along the profiles. The carbon (δ^13^C) and nitrogen (δ^15^N) stable isotopes and elemental analyses (total organic carbon [TOC] and total nitrogen [TN]) were performed at the Stable Isotope Laboratory of the Center for Nuclear Energy in Agriculture (CENA/USP), using an ANCA SL 2020 mass spectrometer. The δ^13^C and δ^15^N values are expressed in parts per mil (‰) relative to the Vienna Pee Dee Belemnite (VPDB; for C) and atmospheric air (for N) international standards, with an analytical precision of ±0.1‰ and ±0.2‰, respectively.

The interpretations about sources of OM were based on previous studies [16,19,47] and references therein. These studies have shown that forest patches typically contribute C₃-derived plant material, which is marked by lower δ¹³C values (approximately –32‰ to –27‰) and a higher C/N ratio due to lignin-rich tissues [17]. On the other hand, rupestrian savannas – adapted to nutrient-poor, rocky substrates – frequently comprise a combination of C₃ and C₄ species, resulting in elevated and non-homogenous δ¹³C values (closer to –32‰ to –23‰) and C/N ratio as shaped by their shrub-herbaceous characteristics [16]. Considering δ^15^N interpretation, nitrogen accumulates along the food chain, leading to lower amounts in plant tissues and higher δ¹⁵N values in predators such as hematophagous and animal-feeding bats [48,49].

To aid interpretation, we compared samples from different environments. The variations in δ¹³C, C:N ratio, and OM composition between forests, lakes, swamps and rupestrian savanna were also investigated to identify ecosystem-specific patterns. For instance, lake sediments often reflect aquatic sources, while forest soils and leaves show more diverse C₃ plant inputs. This comparison clarifies how different environments contribute to the regional organic matter pool.

### Bayesian mixing model (MixSIAR)

The Bayesian isotope mixing model (MixSIAR, version 3.1.7), an open-source R package was used in the estimation of sediment source apportionment [50]. MixSIAR estimates the proportion of each source in the target samples taking into account concentration dependence, variability in source isotopic values and various effects (random, fixed and continuous) on the variability of sediment isotopic composition [51,52]. As input parameters, δ^13^C values and C:N ratio from soils and plant leaves (a total of 471 samples) from rupestrian savanna, grasslands, forest patches, and swamp fields were obtained from the literature [16,19,28,47,53–57] and through the Instituto Tecnológico Vale database. In particular, δ¹³C values and C:N ratio were measured from soil (0–10 cm) and leaf samples of forest patches, and from surface sediment samples (0–10 cm) in lakes (see sampling details in [19,53,54]). Moreover, leaf samples of the most representative vascular plants in poorly drained depressions, rupestrian savannas, and grassland areas were collected to obtain δ¹³C values and C:N ratio [19]. These categories were selected to encompass the very diverse landscapes in the study area, contributing diverse types of organic matter. The forest samples are possibly harvested from a diversity of tree species, whereas lake sediments account for a variety of aquatic and terrestrial sources. In contrast, leaf samples from rupestrian savanna and grassland ecosystems provide valuable insights about organic matter contributions from more open, shrub-herbaceous habitats. The Markov Chain Monte Carlo (MCMC) parameters in MixSIAR were fixed to “long”, and the Gelman-Rubin and Geweke diagnostic tests (Gelman-Rubin statistic <1.05 and for Geweke, low absolute z-score) were applied to assess if the output were adequate [50]. Contributions from distinct sources were analyzed for both average and median values.

To account for differences in sample categories, the MixSIAR model was assessed for sensitivity [58,59] to variations in inputs from distinct geoenvironments. This evaluation involved testing the model’s response to changes in the proportional contribution of OM from different geoenvironments (forest patches, upland lakes, rupestrian savanna, and poorly drained depressions). By using this method, we tested model robustness across several different types of samples, and whether an isolated geoenvironment has a preponderant effect on the model predictions. Addressing these differences allowed us to better identify the sources of organic material and ensured that the results accurately reflect ecological conditions.

### Geochemical data

Downcore elemental variations for the cores SB_0064 and S11B_0168 were measured by an X-ray fluorescence core scanner (Tracer III-SD), on the split core surfaces, at the Center for Nuclear Energy in Agriculture (CENA/USP). The cores were covered with a high-purity polypropylene film and then scanned with 1 cm resolution and a rhodium X-ray source. Runs were conducted at 35 kV, 7 µA, and 15 sec exposure time. The results represent the measured elemental intensities expressed in counts per second (cps) for the elements P, S, Ti, and Fe.

The elemental variations in the profile of cave S11C_0041 were measured with a mini silver anode X-ray tube (Amptek) and a fast SDD detector with 125 eV FWHM for the 5.9 KeV Mn line. Measurements were conducted at 30 kV voltage, 5 μA current, and for 300 s at the Institute of Physics (IF/USP). For each analyzed interval (22 in total), approximately 1 g of dried and ground sample was collected, ensuring sufficient material for detection by the instrument. Data were then processed using WinQxas software and expressed in counts per second (cps) of elemental intensities for the elements P, S, Ti, and Fe. Geochemical analysis was not performed on the S11B_0094 cave profile.

In this study, we focus on the variations of Fe, Ti, and P due to their specific applications as indicators of detrital input (Fe, Ti) and guano deposition marker (P). Fe is commonly associated with minerals such as hematite and goethite and can be carried through physical weathering and erosion of iron-rich rocks in the catchment area [53], while Ti, primarily found in resistant minerals namely anatase, ilmenite and rutile, becomes an effective indicator of terrigenous inflow because of reduced influence of diagenetic alteration [60,61]. Relative abundances of these elements tell us about sedimentary input and composition of source area. In contrast, P is one of the main indicators of guano input in caves as it is introduced primarily by biological activity rather than physical weathering [62]. Guano is abundant in phosphates produced by the digestion of organic matter, which increases its P-enrichment, corresponding to its presence in cave deposits, typically reflecting bat colonies [62].

### Statistical analysis

Descriptive statistics (average, median, minimum, maximum, standard deviation and variance), and Spearman correlation were included in the statistical analyses. The Spearman rank correlation coefficient (ρ) indicates the strength and direction of the relationship between two variables [63], where the magnitude of the correlation ranges from very weak (0–0.19) to very strong (0.8–1.0). Since the dataset contains proportional data constrained to a constant sum, the analysis involved a centered log-ratio (CLR) transformation following the principles of compositional data analysis. Heatmap visualization and Principal Component Analysis (PCA) were subsequently conducted on the CLR-transformed data using R (version 4.3.1) to explore compositional differences among cave samples. Additional graphics were performed using PAST for Windows, version 4.11.

## Results

### δ^13^C values and C:N ratio in cave deposits

**Fig 5:**
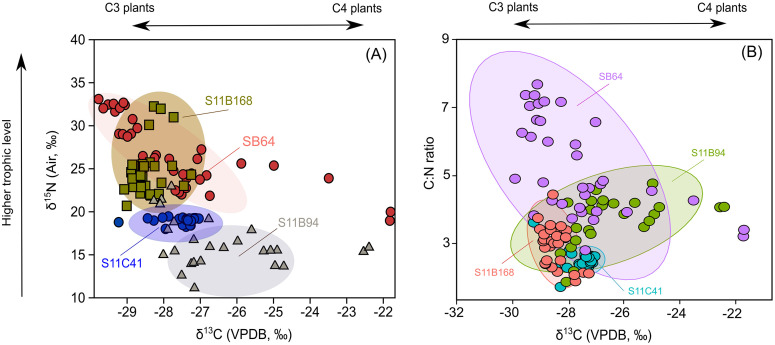
Biplot ofδ^13^C values (‰, VPDB) vs. δ^15^N values (‰, Atmospheric N_2_) (A), and δ^13^C values (‰, VPDB) vs. C:N ratio (B) of cave deposits collected in the Carajás region, southeastern Amazonia.

All δ^13^C values in bat guano samples vary from –29.9‰ to –21.6‰ (Fig 5a), with a mean of –27.6 ± 3.4‰ (n = 168). Minimum mean δ^13^C values are found in SB_0064 (–29.9‰), followed by S11C_0041 (–29.2‰), S11B_0168 (–29.0‰), and S11B_0094 (–28.2‰). Meanwhile, maximum mean δ^13^C values are found in SB_0064 (–21.6‰) and S11B_0094 (–22.3‰). All δ^15^N values vary from 11.9‰ to 33.0‰ (Fig 5), with a mean of –19.3 ± 4.9‰ (n = 168). The lowest mean δ^15^N values are found in S11B_0094 (11.2‰), followed by S11C_0041 (18.0‰). Meanwhile, maximum mean δ^15^N values are observed in SB_0064 (18.9‰) and S11B_0168 (20.6‰).

The C:N ratio in bat guano samples range from 1.7 to 7.6 (Fig 5b), with a mean of 3.4 ± 1.2 (n = 168). The lowest C:N ratio are observed at S11C_0041 (1.7), S11B_0094 (1.8) and S11B_0168 (1.8), while maximum values are found in SB_0064 (2.8). The complete isotopic and elemental data are available in S1 Table.

### Statistical data

**Fig 6:**
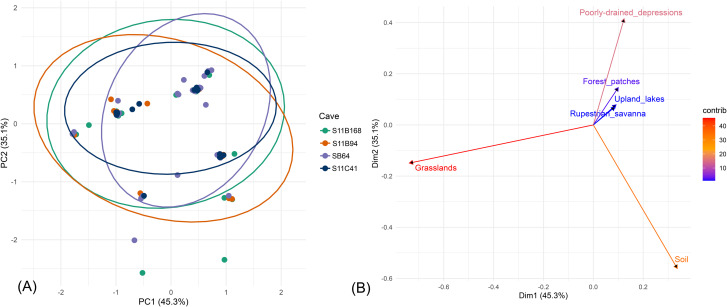
Multivariate analysis of cave samples. **(A)** PCA showing the distribution of samples from four caves (S11B_0168, S11B_0094, SB_0064 and S11C_0041) along the first two principal components (PC1 and PC2), with ellipses representing 95% confidence intervals. **(B)** Environmental vector fitting on the PCA ordination indicating the direction and strength of correlations between geoenvironmental variables (arrows) and the sample distribution. The color gradient of the arrows reflects the contribution of each variable to the ordination.

The PCA in the compositional data (after CLR transformation) indicated that the first two axes explained 80.4% of the full variance, with PC1 explaining 45.3% and PC2 for 35.1% (Fig 6a).

Samples from the four caves showed a close matching within the ordination space, with 95% confidence ellipses that overlapped and that did not provide an obvious multivariate separation among caves. The pattern indicates that the proportional contributions of the categories are mostly the same in caves, where the greatest variation is found within caves rather than between caves. On the PCA biplot, PC1 was almost exclusively defined by a pronounced contrast between grasslands and soil (Fig 6b). Grasslands showed a strong negative loading for PC1 and contributed greatly to this axis, whereas soil exhibited a strong positive loading, showing an inverse relationship between them along the main gradient of variation.

In contrast, PC2 was primarily affected by poorly drained depressions, with considerable positive loading and large contribution to this axis. Soil also played a role in PC2, but it was in the opposite direction, reinforcing its contrasting role across the two principal components. The forest patches, doline-shaped lakes, and rupestrian savanna appeared to make lower contributions to both axes, forming clusters near the origin of the biplot. Therefore, these categories made a weak contribution to the overall variance captured by the first two components and varied more subtly among samples. Altogether, the PCA suggests that variation in landscape composition among samples is dominated by the opposing contributions of grasslands, soil, and poorly drained depressions, while the remaining categories show relatively stable proportional contributions across caves.

### Geochemical data

**Fig 7:**
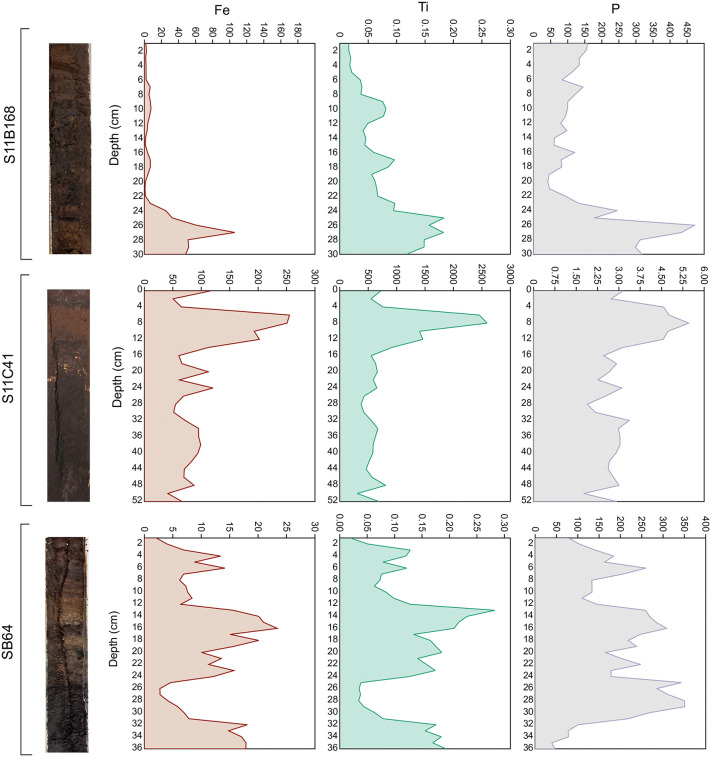
Variation in elemental intensities of Fe, Ti, and P along the profiles of cave deposits from S11B_0168, S11C_0041, and SB_0064.

The variations in Fe, Ti, and P intensity along the profiles of caves S11C_0041, S11B_0168, and SB_0064 show distinct patterns (Fig 7). In S11B_0168, Fe and Ti show a gradual decrease toward the top. According to the Spearman rank correlation coefficient, these elements are moderately correlated (ρ 0.56), while P presents marked oscillations, with distinct peaks and lower intensity in between, and very weak correlation with Ti (ρ 0.09). On the other hand, S has a strong correlation with P (ρ 0.66). In S11C_0041, Fe and Ti maintain relatively stable intensity with moderate fluctuations throughout the profile and a peak at the top (between 4 and 16 cm). These elements are still moderately correlated (ρ 0.56) in this profile. P exhibits a similar pattern, with a well-defined peak at the top (4–16 cm). Indeed, P and Ti are strongly correlated (ρ 0.70), while very weakly correlated to S (ρ 0.15). In SB_0064, Fe and Ti display similar patterns and strong correlation (ρ 0.81), with abrupt variations across the profile. P remains at a high intensity throughout most of the profile and shows very weak correlation coefficient with Ti and Fe (< 0.02), but strong correlation with S (ρ 0.66).

### Contribution of allochthonous material in cave deposits

**Fig 8:**
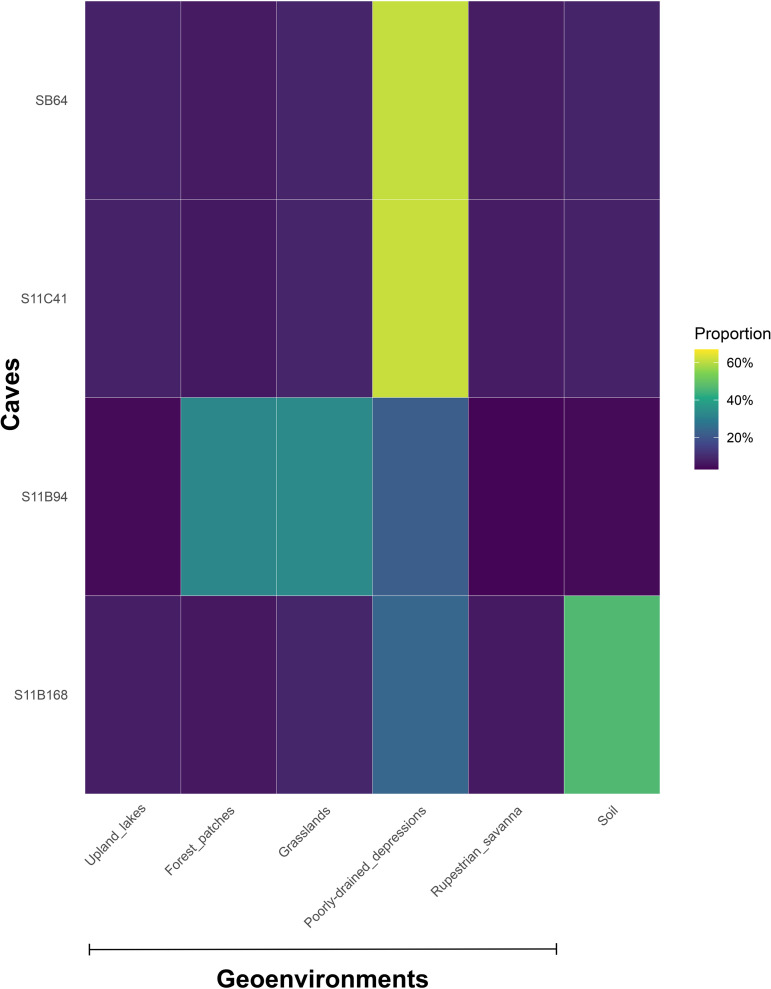
Heatmap showing the proportional contribution of different geoenvironmental categories (grasslands, forest patches, rupestrian savanna, poorly drained depressions, and upland lakes) to each cave. Colors represent relative proportions (from low to high), highlighting differences in landscape composition among caves.

The Bayesian mixing model indicated a large contribution of poorly drained depressions (median: 41.5–42.9%) and grasslands (median: 8.8–15.1%) to the four cave deposits (Fig. 8; Table 3). Smaller contributions were estimated for soil (median: 8.3–8.4%), upland lakes (median: 6.8–7.8%), rupestrian savanna (median: 6.2–6.6%), and forest patches (median: 5.9–6.5%) (S2 Table).

**Table 3:** The median contribution values for each category (upland lakes, forest patches, grasslands, poorly drained depressions, rupestrian savanna, and soils) across the studied caves in Carajás Region, southeastern Amazonia. The highest values are highlighted in bold.

Cave ID	Categories					
	Upland lakes	Forest patches	Grasslands	Poorly drained depressions	Rupestrian savanna	Soils
S11B_0168.	6.8%	6.3%	**9.1%**	**41.5%**	6.2%	8.3%
S11B_0094.	7.0%	5.9%	**15.1%**	**41.7%**	6.2%	8.3%
S11C_0041.	7.8%	6.4%	**8.8%**	**42.9%**	6.6%	8.3%
SB_0064	7.7%	6.5%	**9.0%**	**42.6%**	6.6%	8.4%

The heatmap depicts clear differences in the proportional contribution of geoenvironmental categories among caves. The contribution of OM from poorly drained depressions predominates in SB_0064 (3.3–62.3%) and S11C_0041 (23.2–61.5%), representing the largest share of the surrounding landscape in these caves. In contrast, S11B_0094 reveals a more diverse composition, with a high contribution of OM from poorly drained depressions (21.9–61.7%) and grasslands (7.7–60%), along with a moderate contribution from forest patches (3.9–32.6%). From the other caves, S11B_0168 shows a greater proportion (5–67%) of soil in this cave (the vast majority) than for the others.

## Discussion

### Primary sources of allochthonous material in cave deposits

Each sampled environment possesses distinct ecological characteristics that shape the composition of OM. Forests (dense vegetation and complex canopy structure) produce OM with isotopic signatures that differ from savanna and grasslands, the latter being those of more open ecosystems. Lake sediments, however, provide OM composed of a combination of allochthonous and autochthonous OM derived from terrestrial and aquatic sources, respectively. Such ecological variations play a key role in determining the carbon isotopic signature of the OM and its nutrient compositions, both significant considerations in the interpretation of model outcomes.

The swampy fields and grasslands – the geoenvironmental units within the landscape compartment of poorly drained depressions (see Table 1) – constitute major contributors to the deposit of OM in caves. These geoenvironments are seasonally water-retaining systems; they aid in the storage and transport of organic and inorganic material. Their contribution to OM input is likely due to water-mediated transport through fractures and seepage [64]. This underground flow may explain why some caves (e.g., S11B_0094 and S11C_0041) exhibit persistent humidity, showing that there is dripping and pooled water even in the dry season.

Moreover, the presence of caves located on the slopes of the Carajás plateau [65], where the surface is characterized by broad poorly drained depressions (see Fig 1c), indicates that a large part of OM present in cave deposits likely originates from underground flow. Percolation and infiltration are processes that allow organic material to move from this landscape compartment into subterranean environments. Grasslands are strongly influenced by hydrological dynamics, being prone to flooding in the rainy season and a notable decrease in water-covered areas in the dry season. In contrast, swampy fields are marked by permanent flooding in the depressions of the terrain. Surface cover in these accumulation areas favors the formation of “suspended aquifers” or permanently saturated zones. Like lakes, the erosion and weathering at the higher slopes of the land leads to greater accumulation of clay in the depression, which in turn increases water retention. While lakes constitute an important source of water for underground percolation, the deposition of sediments more than 10 meters thick in the lacustrine basin likely contributes to the impermeabilization of the depression [64,66].

The environment and type of deposits of OM in caves are also likely to represent an additional factor leading to the composition of the surrounding landscape. Most caves in Carajás are located in or near wide grasslands and shrublands or transitional zones between rupestrian savanna and forest formations, probably leading to a higher proportion of grassland-derived material in the deposits. Seasonal variation and resource availability are critical factors as well. There is a sudden boom in insect populations in open habitats during the rainy season [67]. In addition, the appearance of ephemeral lakes and swampy fields, which are often grass-dominated, might lead to bat foraging in these areas. Accordingly, OM derived from grassland deposits may be a more prominent component in cave deposits during periods of insect abundance.

Soil organic matter is probably transported by erosion of the deeper soils that are developed under the forest patches and introduced into caves by water runoff. Based on the results from the MixSIAR model, terrestrial sources such as forest patches contribute minimally to the overall OM composition in cave deposits. Their contribution is limited due to their restricted spatial distribution throughout Serra Sul and their low occurrence near the studied caves (see Fig. 1c), resulting in a diluted signal compared to other geoenvironmental units (e.g., grasslands).

On the other hand, the open ombrophylous forest, a phytophysiognomy found at the entrance of caves, can potentially influence organic matter composition by producing a large volume of OM, mainly by shedding leaves, branches, fruits, and other plant debris. Although this phytophysiognomy is not included in the present study, its litter can be transported into caves through wind, surface runoff, or water percolation, serving also as a food resource for cave-dwelling fauna [68,69]. Frugivorous and nectarivorous bats that eat fruits and nectar from forest areas and cohabit the caves with insectivorous bats (e.g., *Lonchorhina aurita*) preferentially use a forested habitat [69] and their material can be incorporated into the guano for the formation of a composite forest-derived organic material, as observed in cave S11B_0094 (see Fig 8).

The changes in OM input in caves are also likely driven by the foraging strategies and dietary factors among bat species, which differ from one taxon to another [70–72]. In this sense, δ¹⁵N values serve as a useful unit of trophic level proxy, as high δ¹⁵N values are more usually indicative of insectivorous diets while lower δ¹⁵N values tend to be associated with nectarivory and frugivory [48]. Still, prior studies suggest that nectarivorous bats can have higher δ¹⁵N values than the strictly frugivorous bat species [49]. This behavior is possibly due to dietary flexibility of nectar-feeding bats who are relatively nectar- and pollen-eating as well as eat insects opportunistically [73], which in turn involves nitrogen from upper trophic levels, with relatively enriched δ¹⁵N values. Based on that, caves SB_0064 and S11B_0168 predominantly colonized by insectivorous bats have relatively higher δ¹⁵N values, which may reflect higher organic matter inputs from the upper trophic levels (Fig. 5a).

Conversely, caves S11B_0094 and S11C_0041, mostly populated by frugivorous and nectarivorous bats, exhibit relatively low δ¹⁵N values, suggesting contributions from lower trophic levels and primary production-based food webs. These dietary differences are also related to habitat use, as bats belonging to the insectivorous feeding guild, including members of the family Molossidae, typically forage in the open-air environments above the forest canopy [74–76]. Meanwhile, frugivorous and nectarivorous bat species are likely to prefer forested or edge habitats, and the more depleted δ¹⁵N values in the cave-deposited organic matter of the two sources are also evident, in line with their generally lower trophic position of dependency or relative reliance on plant-dominated resources.

### The input of detrital material into caves

For cave S11C_0041, Fe and Ti have relatively stable variations with fluctuations across the profile (see Fig 7). This moderate correlation points to a detrital origin of the material [61], which can be allochthonous (for instance, lateritic crust) or sourced from detrital minerals of the cave’s host rocks [77]. The strong correlation of Ti with P suggests phosphorus was introduced into the cave with detrital particles most likely originating from the weathering of the canga [78]. The weak correlation between P and TOC, supporting the assertion that phosphorus input is linked predominantly to mineral transport rather than organic material input, helps also to suggest some of the processes involved. Phosphate minerals can be produced from the interaction of guano derived solutions with the cave bedrock, or with secondary cave deposits (either chemical or detrital) [79].

Fe and Ti are moderately correlated in cave S11B_0168 — this reinforces their detrital origin. Gradually increasing depths, at a maximum of 24–30 cm, suggest a phase of enhanced detrital input. Also, the low correlation between P and both Fe and Ti suggests that the majority of P is obtained from organic sources. This also supports fluctuations of P levels which further suggest periodic changes in guano deposition, likely driven by variation in biological activity in the cave.

Likewise, in cave SB_0064, Fe and Ti also closely correlate with one another. Irregular patterns are present throughout them and the whole profile changes suddenly, which is likely to be attributable to differing periods when detrital input occurred. Whereas the very weak relationship between P and both Fe and Ti suggests that phosphorus originates principally from organic sources. This pattern may indicate that the cave functions as a maternity roost, where large bat colonies, primarily composed of females and their offspring, are present for extended periods of the year [80,81]. These caves normally gain more balanced input from organic matter over a longer interval. In contrast, other explored caves may function as satellite roosts that experience large seasonal fluctuations in bat abundance (including periods of prolonged abandonment) and are mainly used for mating [80].

These profile differences illustrate the extent to which organic and inorganic inputs and accumulation varied and were driven by ecological diversity, depending on the specific ecological dynamics of each cave. Whereas some deposits show strong biological signals (marked P peaks), others are dominated by sedimentary inputs, reflected by high Fe and Ti intensity that indicate episodes of detrital influx interspersed with guano deposition).

### Environmental and geological drivers of cave similarities and differences

The similarities observed in OM sources between cave deposits are related to a similar geological setting and regional geomorphological context. All caves are developed within the same ironstone plateau, characterized by lateritic crusts, shallow soils, and a mosaic of open and forested geoenvironments [33]. This shared geological framework limits hydrological pathways, sediment availability, and erosion processes, allowing similar sources of organic and mineral inputs in caves [14]. As a result, even though the relative contributions of various geoenvironmental units vary throughout sites, they are consistently represented.

On the other hand, differences between caves seem to be predominantly determined by a local-scale variability in surface geoenvironmental composition – especially whether poorly drained depressions, soil cover, or vegetated units dominate these areas. Caves surrounded by poorly drained depressions receive proportionately larger inputs from waterlogged environments – a condition favoring the accumulation and preservation of organic matter. In contrast, caves embedded in landscapes with greater soil exposure or mixed vegetation units reflect a more heterogeneous input signal, integrating materials derived from multiple surface sources.

The hydrological network between underground and the exposed surface is likely to be a key factor in structuring these patterns. In poorly drained depressions, dissolved and particulate material can move to the caves in an efficient manner during raining episodes; in relatively well drained or vegetated places, direct runoff may be minimized and slow infiltration may occur. These processes are then further modulated by slope, microtopography, and drainage density around each cave, which impact the amount and type of material deposited within cave systems.

Finally, the interplay between geoenvironmental composition and biological activity supports cave-specific signatures. Changes in surface vegetation structure and soil development influence not only sediment flow, but also the supply (and the composition) of organic material available to cave fauna, including bats. In fact, the apparent differences between caves are expected to be attributed to the effects of local geomorphology, hydrological dynamics and landscape heterogeneity, which were all superimposed on a common regional geological setting. These factors combined create unique but similar depositional settings within the cave systems.

### Broader applicability and methodological implications of the approach

In addition to the site-specific interpretations, the integrative framework used in this study has methodological implications for cave research at large. Through integration of geoenvironmental mapping with compositional data analysis and multivariate methods, this framework enables the measurement of surface–subsurface connections quantitatively, rather than inferring them qualitatively. This is an improvement over the usual cave research, which largely centers on isolated proxies, as it explicitly accounts for the relative contribution of multiple surface units to cave deposits.

Compositional techniques, including MixSIAR, are particularly applicable to environments where inputs of sediment and organic matter are inherently constrained to relative proportions [82]. Conditions like this are common not only in ironstone cave systems but also in karst, pseudokarst and shallow subterranean environments worldwide. Thus, the methodological approach proposed here can be easily applied in other locations where caves combine signals from diverse surface terrains, such as carbonate karst terrains, and lateritic or ferruginous systems, allowing for comparisons on both spatial and temporal scales. Where geoenvironmental contributions are expressed as proportions, data from distinct cave systems or geological substrates can be compared against one another allowing for regional syntheses and cross-system assessments. Furthermore, combined with geochemical or biological proxies (e.g., stable isotopes or pollen assemblages), this framework offers a robust tool for disentangling the relative influence of geomorphology, hydrology, and ecology on cave depositional processes.

Overall, the proposed framework provides a generalized, flexible approach for exploring the relationship between cave–landscape interactions. More importantly, its emphasis on proportional representation, statistical robustness, and the integration of surface and subterranean processes makes it well suited for advancing cave-based research beyond local case studies toward broader comparative and process-based investigations.

## Conclusion

This research reveals that deposits in the iron-ore caves of Carajás are largely driven by input from allochthonous material derived from surface geoenvironmental units, including poorly drained depressions. The findings showed that cave environments are not isolated systems but provide natural service in the surrounding landscape where organic matter enters either indirectly through hydrological infiltration or biologically by means of bats. The ecological significance of these caves lies in their function as repositories of surface-derived organic matter, and as bridges connecting surface ecosystems and the subterranean food web.

From a conservation perspective, our findings highlight the necessity of preserving cave ecosystems beyond their bounds. The conservation of the surrounding landscapes, including wetlands, lakes, and vegetation mosaics that regulate water flow and support bat populations, is critical for the ecological integrity of cave systems. Furthermore, the integrated approach used in this study provides a framework for quantifying surface-subsurface connectivity in different karst and pseudokarst ecosystems globally. This approach, which combines geoenvironmental mapping with compositional and multivariate analyses, can support comparative research and guide conservation strategies for underground biodiversity in the face of growing environmental challenges.

## Supporting information

S1 Table Isotopic and elementar dataset. Carbon and nitrogen stable isotope values (δ¹³C relative to VPDB and δ¹⁵N relative to atmospheric air; ‰) and elemental composition (C and N; %) of guano deposits from the Carajás region southeastern Amazonia.
(XLSX)

S2 Table MixSIAR dataset. Proportional contribution values for each category (upland lakes, forest patches, grasslands, poorly drained depressions, rupestrian savanna, and soils) across guano profiles from the studied caves in Carajás region, southeastern Amazonia.
(XLSX)
